# Common bottlenose dolphin (*Tursiops truncatus*) behavior in an active narrow seaport

**DOI:** 10.1371/journal.pone.0211971

**Published:** 2019-02-19

**Authors:** Sarah Piwetz

**Affiliations:** Department of Marine Biology, Texas A&M University at Galveston, Galveston, Texas, United States of America; Hawaii Pacific University, UNITED STATES

## Abstract

The Galveston Ship Channel (GSC) is a narrow, congested waterway that supports large-scale shipping, commercial fishing, dolphin tourism, and recreation. Human activity and common bottlenose dolphins (*Tursiops truncatus*) converge in the GSC with potentially negative consequences on the dolphins. Elevated land-based tracking and behavioral observation of dolphins and vessels were conducted along the GSC in June-August 2013 using a digital theodolite. Positional information was used to calculate dolphin movement patterns and proximity to vessels. Log-likelihood ratio and Chi-square contingency tests were used to assess behavioral states, and generalized additive models were used to analyze movement patterns (i.e., swimming speed, reorientation rate, and linearity) relative to endogenous and exogenous factors and vessel presence. Dolphins regularly use the GSC to forage (57% of observed behavioral states) and socialize (27%), and it is not a travel corridor for accessing other favorable sites (traveling = 5%). Dolphin behavior varied significantly based on time of day, group size, calf presence, and general boat presence. When boats were present, the proportion of time dolphins spent socializing and foraging was significantly less than expected by chance. Swimming speeds increased significantly in the presence of small recreational boats, dolphin-watching tour boats, shrimp trawlers, and when tour boats and shrimp trawlers were both present. Reorientation rate increased significantly in the presence of tour boats and trawlers. Dolphin behavioral responses to vessel presence may result in decreased energy consumption due to disrupted foraging activity. Without proper management, the observed behavioral changes may be detrimental to individuals within this population in the short term, with potential long-term consequences to health and survivorship.

## Introduction

Marine mammals that inhabit near-shore waters are exposed to recreational and commercial activities [1, 2] where habitats and resources, such as prey items, are exploited by humans [3]. Disruption to marine mammal habitat-use and behavior have been documented in coastal areas with concentrated human activity including aquaculture farming [4, 5], active and simulated mid-frequency sonar noise [6], dolphin-based tourism [7, 8], and human-induced habitat degradation [9]. Coastal-living marine mammal species are often found in or near natural and artificially-dredged channels where fish prey are abundant or aggregated [10–13]. The ability of dolphins to exploit features of channels, such as steep slopes, may increase their efficiency of prey detection and acquisition by providing barriers with which to herd prey [14]. Dredged channels created for large vessel passage may increase feeding opportunities for dolphins. However, exposure to vessels is intensified in narrow shipping channels (<1km wide) where space available for horizontal movement is limited.

Common bottlenose dolphins (*Tursiops truncatus*, hereafter “bottlenose dolphins”) occur in the narrow and congested artificially-dredged Galveston Ship Channel (GSC), within Galveston Bay, Texas, year-round [15]. However, little is known about their habitat-use and behavior relative to diverse vessel types and activities. A three year study in the early 1990’s photo-identified 240 individuals in the GSC, 75% of which were resighted [16]. Dolphins that use the GSC likely represent an open population with minimal net change in population size and may include dolphins from the overall Galveston Bay strategic stock, delineated by the National Marine Fisheries Service (NMFS), and the western Gulf of Mexico coastal stock [16]. The GSC has the highest percentage of foraging dolphin groups within the Galveston Bay system, and is one of two main feeding areas in Lower Galveston Bay. This may be due, in part, to the deep dredged channels where highly-saline waters and prey species enter from the Gulf of Mexico [17]. Bottlenose dolphin behavior has been studied extensively on a global scale, but the behaviors of populations off Texas have received less attention than those in other areas such as Florida (*T*. *truncatus*) [18] and Australia (*Tursiops sp*.) [19]. Several studies have focused on dolphin habitat-use of deep, narrow channels, but few have examined dolphin responses to diverse vessels types in these active seaports.

The GSC ranks among the top 50 U.S. ports in terms of total tonnage and lies within Galveston Bay, the largest estuary in Texas and seventh largest in the United States (U. S.) [20]. The GSC has the capacity to accommodate large, fully loaded ships and is an important contributor to the regional economy [21]. In addition to the transportation sector, for which it was developed, the GSC supports commercial fisheries (Galveston “Mosquito” Shrimp Fleet), dolphin-watching tourism (3 companies), high-speed amusement boat rides (one company), and private recreational boating and fishing. It is listed as having heavy maritime congestion and the most diverse mix of vessel types within the Houston-Galveston Port area where “no one follows a traffic scheme” [22]. Bottlenose dolphins that utilize this area may potentially tolerate vessel disturbance for access to concentrated prey assemblages [23]. However, the NMFS threat assessment identified boat traffic and tourism among the top 4 of 19 potential threats to bottlenose dolphins in Galveston Bay (including the GSC) [24].

Interpreting wildlife responses to human activity is complex, and behaviors can be influenced by a variety of natural and human generated factors. Many conservation-based studies involving marine mammals and human activity focus on direct interactions that lead to injury or mortality, such as vessel strike, fisheries bycatch, and entanglement [25–27]. Signs of direct injury to bottlenose dolphins are observed in the Galveston Bay region (e.g., fishing gear entanglement, propeller wounds), but it is unclear how these incidents relate to overall mortality (pers. comm. Heidi Whitehead, Texas Marine Mammal Stranding Network, 2017). Anthropogenic activity also affects marine mammals in ways that are less obvious, such as altering behavior [28, 29], and can be detected using behavioral indicators of disturbance [30]. The conservation behavior framework, a parsimonious model that aims to link behavior and conservation, proposes that behavior-oriented conservation studies focus on: 1) human impacts on animal behavior (potential stressor stimuli), 2) behavioral indicators (responses to potential stressors), and 3) behavior-based management [30].

A variety of short-term behavioral responses by odontocetes (toothed whales) including horizontal and vertical movements, have been described relative to marine vessel traffic in other areas that include fleeing, diving for longer durations, and avoiding areas with vessel traffic altogether [31]. Dolphins have also been observed altering grouping patterns or inter-individual distances when vessels are present [32]. Alterations in swimming speed, and reduced or disrupted foraging, resting, and socializing bouts have also been reported in response to vessels, including dolphin-watching tourism [33–42]. Metrics used to identify shifts in behavior include quantifying inter-breath intervals, distance to nearest neighbor, swimming speed, reorientation rate, and linearity. Not all behavioral responses are classified as avoidance, and dolphins may alter behavior to approach vessels. For example, dolphins may be attracted to discarded bycatch, or prey stirred up by, or caught in, the nets of commercial trawlers [3]. These risky attractants, in which prey acquisition may be facilitated, increase the risk of injury to dolphins via propeller lacerations or net entanglement. In the U.S., disturbing a marine mammal’s behavior pattern is considered harassment, as defined by the U.S. Marine Mammal Protection Act (MMPA) of 1972 [43]. Harassment in this context includes any act of pursuit that has the potential to disturb a wild dolphin by disrupting behavioral patterns, including, but not limited to, feeding [43].

In this study, bottlenose dolphin behavior in the GSC was investigated in the absence and presence of diverse vessel activity and provides evidence-based suggestions to balance maritime vessel activities with marine mammal protection. Specific objectives include: 1) determine how dolphin behavioral activity states vary based on endogenous (group size and composition) and exogenous (time of day) factors and vessel presence; 2) quantify movement patterns (swimming speed, reorientation rate, and linearity) to assess dolphin behavioral responses to diverse vessel traffic; and 3) provide science-based recommendations for adaptive management to contribute to conservation efforts in a fluctuating environment.

## Materials and methods

### Ethics statement

Data were collected from shore and no approaches to or harassment of animals or vessels were performed. No permits were required for this fully non-invasive method.

### Study area

**Fig 1:**
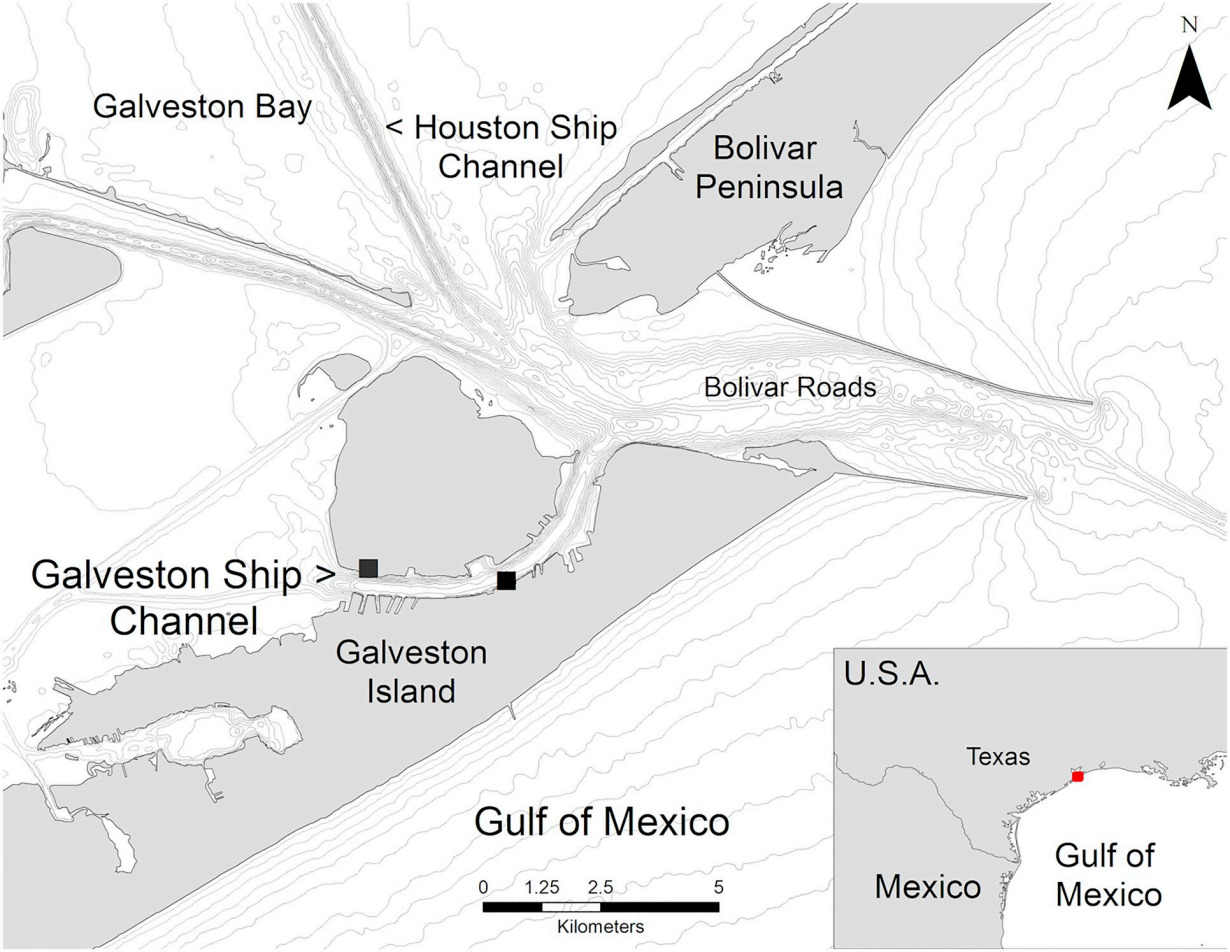
Study area showing theodolite observation platforms (■) and isobaths at 5m intervals. Map created using ArcGIS software from Environmental Systems Research Institute (ESRI) and shapefiles from Texas Natural Resources Information System (Public Domain–Creative Commons CCO).

The Galveston Ship Channel (29° N, 94° W) is located on the upper Texas Coast, at the mouth of the Galveston Bay Estuary, with adjacent access to the Gulf of Mexico via Bolivar Roads (Fig 1). The channel is narrow, between 370m-980m wide, extends 6.8km in length, and has a steep U-shaped human-altered slope with maximum dredged depths of 14m [44, 45]. Bottlenose dolphins are the only marine mammals that are regularly observed in the Galveston Bay area.

### Sampling methods

Elevated land-based theodolite tracking of individual and groups of dolphins and vessels was conducted along the GSC in summer (June-August) 2013. Field observations totaled 31 days and 158 hours on effort. Three observation areas were selected based on close proximity to the water, elevation above sea level, and views of the channel (Fig 1). The viewing area between any two locations overlapped by approximately 20% to 40%. However, only one location was used per day, eliminating potential double sampling of the same dolphin group on the same day. Data collection rotated among locations, with a fairly even sampling schedule of 8 days at location 1, 10 days at location 2, and 11 days at location 3. A digital theodolite (Sokkia/Sokkisha Model DT5) with 30-power magnification and ±5-second precision was used to obtain vertical and horizontal angles of dolphin and vessel positions [46]. *Pythagoras* software (v 1.2) [47] was used to record and convert theodolite angles to geographic coordinates, record surface behavioral states, and facilitate data management for analyses. Systematic scans of the viewable area at the start of each tracking session to locate dolphins were conducted with hand held binoculars (7x50 magnification). Data collection involved three team members—a theodolite operator, an observer, and a data-entry computer operator. To minimize inter-individual variation in data gathering, theodolite tracking was conducted by the same experienced operator (Piwetz) at all times. The observer and computer operator rotated positions every hour to reduce potential for visual fatigue.

Dolphin groups were defined using a combination of the 10m chain rule (individuals within 10m of another individual are part of the same group) [48] and coordinated activity [49]. Focal follow [50, 51] sessions were initiated once dolphins were located. Often, only a single individual or group was present, making focal group selection straightforward. If multiple groups were present during times of high vessel traffic, only one group was designated for the focal follow. To reduce bias in group selection, the larger group was selected for the first session, the smaller group for the subsequent session, and so on [51]. During low vessel traffic, multiple individuals or groups were followed simultaneously. If group members split during a follow, an attempt was made to alternate between following the smaller group the first time members split, the larger group if members split again, and so on.

Data were recorded continuously and subsampled at 60 sec intervals *post hoc*. Focal dolphin data included geographic position, group size, calf presence/absence, and predominant group (≥50% of individuals) behavioral state. Age classification included adults and calves only, excluding subadults given the difficulty in accurately distinguishing this class consistently. Calves were classified based on size (2/3 the length of an adult, or less) and swimming position (echelon position “beside and slightly behind an adult”) [52]. Surface behavioral states were classified using a combination of definitions (Table 1). Focal individuals were tracked by fixing theodolite crosshairs on the animals’ body at the water line. Groups were tracked by recording positions based on a central location within the group [53, 54]. Focal follows continued until the individual or group was lost, moved beyond the range of reliable visibility (>3.5km), or environmental conditions obstructed visibility (e.g., intense haze or fog, Beaufort sea state >3, or sunset).

**Table 1:** Bottlenose dolphin behavioral state definitions.

Behavioral State	Definition	Source
Foraging	Variable direction of movement, generally remaining in the same area, high arching dives, fish chasing or tossing, little apparent interaction between individuals	[55]
Foraging in association with shrimp trawlers (FSB)	Repeated dives in varying directions around the side or behind the stern of shrimp trawlers	[15]
Resting	Moving very slowly or drifting in one direction	[52]
Socializing	Variable direction of movement, individuals in close proximity or touching, often interacting, frequent surface active behavior	[55]
Traveling	Moving steadily or rapidly in one direction Often synchronous and frequent surfacings	[52] [55]

All vessels that moved within approximately 500m of the focal individual/group were tracked via theodolite. Vessel data were recorded continuously, alternating with dolphin data, and included geographic position, vessel type, vessel name (if available), and activity (e.g., travelling, stationary, following dolphins). Due to small sample size, vessels were broadly categorized *post hoc* based on vessel type, vessel length and movement characteristics (Table 2).

**Table 2:** Vessel categories based on size and movement characteristics.

Category	Vessel types	Vessel length	Mean speed, km/hr (SD; Max)	Mean rr, deg/min (SD; Max)
Small	Personal recreational watercraft Commercial amusement ride	<10m	35.31 (18.57;78.94)	34.10 (42.09; 178.19)
Mid	Government boat (e.g., USCG) Harbor pilot boats University research vessel Tug boat (single, unattached) Yacht	10-30m	14.34 (7.44; 42.85)	18.96 (29.68; 133.01)
Large	Barge (w/ tug boat attached) Cargo ship Cruise ship	>30m	9.19 (4.58; 21.68)	19.82 (33.83; 166.17)
Tour Boat	Dolphin tour boats	9-16m	9.42 (5.74; 49.01)	41.38 (48.50; 178.19)
Trawler	Galveston shrimp trawler fleet	Variable	3.99 (3.01;42.14)	37.75 (45.09; 178.45)

A Before-After-Control Impact (BACI) experimental design is often used to monitor effects of variables over time by comparing responses in a treatment area with a control area [56, 57]. This design was not possible due to the lack of an ecologically similar adjacent site utilized by dolphins, with no vessel traffic, to that of the deep and narrow GSC. Likewise, a Before-During-After (BDA) experimental design, often used to monitor variables over time within the same site, was not logistically feasible [57]. Vessels could not be experimentally controlled in this heavily saturated and economically important shipping port. Therefore, a natural variation of the BDA design was used by collecting data in the presence (test) and absence (control) of the potential stressor (opportunistic vessel approaches) within the same area [57] and the multivariate generalized additive modelling (GAM) framework was applied to control for confounding effects [58].

Dolphins were observed every day of data collection, totaling 278 groups tracked via theodolite, with nearly 6,000 data records. Data were filtered to exclude dolphin tracks with less than 10 min in duration (14.82% of raw data), and/or erroneous data with swim speed exceeding known maximum values (28-40km/h) [59] for bottlenose dolphins (0.7% of raw data). For standardization among observations that varied in duration, each focal follow was binned into multiple 10-min segments [36, 60], comprising 11 interpolated positional fixes per segment (no data points were double counted), with associated data, based on 60 sec intervals. The time interval was selected to avoid errors associated with non-linear travel, established in other marine mammal movement studies [54, 60, 61]. Of the 278 groups tracked, 167 10-min segments from 89 focal follows met the criteria for analyses. The number of segments per focal follow varied based on duration (e.g., a group followed for 30 min would have 3 segments, a group followed for 40 min would have 4 segments). Because successive observations of animal movements pose problems due to lack of independence, temporal autocorrelation was performed during preliminary analysis to identify potential pseudo-replication [62] for groups with more than one 10-min segment.

Dolphin response variables that were calculated for each 10-min segment included mean swimming speed (how fast dolphins swim), reorientation rate (degree of bearing changes per minute), and linearity (an index of net movement) [54, 63, 64]. Swimming speed (km/hr) was calculated by taking the distance travelled and dividing by the duration between two consecutive dolphin positions, and calculating the mean for each segment [65]. Reorientation rate (degrees/min) was calculated by taking the sum of bearing changes within a segment and dividing by the total duration of that segment. Linearity is an index of deviation from a straight line ranging from 0 to 1, with 0 representing no net movement and 1 representing moving in a straight line. Linearity was calculated by dividing the net distance between the first and last fix of a segment by the sum of all distances travelled between each of the 11 interpolated positional fixes within a segment. Response variables were transformed (Log10 for swimming speed, Square Root for reorientation rate, and Empirical Logit for linearity) to approximate a normal distribution.

Candidate explanatory variables for each segment included time of day, dolphin group size, calf presence/absence, predominant dolphin behavioral state, number of vessels present, and type of vessels present. Sunrise and sunset varied throughout the summer season, so a time of day index was calculated to represent a percentile of daylight hours where sunrise = 0 and sunset = 1. Time of day was then broadly categorized into morning (0–0.33), mid-day (0.34–0.66), and afternoon (0.67–1). Dolphin group size was separated into five categories: singletons, dyads, 3–4 individuals, 5–6 individuals, and 7+ individuals. Issues of collinearity among potential explanatory variables were assessed for potential masking effects via augmented pairs plots (correlation coefficients >0.60) and Variance Inflation Factor (VIF) values (i.e., no values >10) [66, 67]. If collinearity was expressed among a pair of explanatory variables, the most interpretable variable was preserved, and the other variable was dropped (S1 Fig). The 10-min segments calculated for movement analysis were also used to analyze behavioral state data. However, all 11 data points within each segment were considered, totaling 1,837 records, due to potential fluctuating behavior within each segment.

Dolphin movement patterns, when no vessels were present, were analyzed to establish a control. The NMFS Southeast Region suggests that vessels maintain a minimum distance of 45m from dolphins [68]. For this study, vessels were considered “present” when traveling within 100m of the focal individual/group. The distance threshold encompasses the NMFS suggested distance of at least 45m and extended to 100m [69] to include dolphins that were actively following behind shrimp trawlers (hereafter ‘trawlers’). The “no vessel” category included dolphin tracks for which no moving vessels were present during and for at least 10 min prior to the focal follow to reduce the potential that dolphin movement was influenced by recent vessel presence.

### Statistical analysis

The following univariate and multivariate statistical analyses were conducted: Chi-square contingency and log-likelihood ratio tests [70] to assess nominal categorical data (i.e., behavioral states), Freeman-Tukey Deviate and binomial *z* score [71, 72] post hoc tests to assess which factors occurred more or less frequently than expected by chance, and GAMs [58] to evaluate continuous numerical data (i.e., movement patterns). Chi-square and Freeman-Tukey Deviate tests were performed for behavioral state (i.e. an array with a single variable), and log-likelihood and binomial *z* score tests were performed for arrays with multiple variables (i.e., behavioral state based on time of day, group size, calf presence, and vessel presence). The Freeman-Tukey Deviate *z* score decision rule was based on the critical value 0.95, and the binomial *z* score decision rule was based on the critical value 1.96. Group size categories were pooled together to meet expected frequency requirements (minimum expected value >5).

Linear mixed-effects modelling was run to detect autocorrelation, using the lme function (package nlme) in program R (v 3.2.2). The fully saturated linear mixed-effects model incorporated fixed effects of time of day and vessel category, and the random effect of successive segments from a single dolphin focal group. The best fitting model for swimming speed included the fixed factor of vessel category, and no significant autocorrelation was found in the residuals (S2 Fig). The models including both fixed factors did not show significant autocorrelation either. The best fitting model for reorientation rate and linearity included an interaction between both fixed factors, and no significant autocorrelation was found in the residuals (S2 Fig).

The GAM framework was applied to relate dolphin movement patterns (i.e., swimming speed, reorientation rate, linearity) to five candidate explanatory factors: time of day, group size, calf presence, predominant group behavioral state, and vessel category present. The candidate explanatory variable ‘vessel number’ was dropped from the fully saturated model to address issues with multicollinearity. No significant collinearity was detected among remaining candidate explanatory variables, based on augmented pairs plots and VIF values. The fully saturated GAM also incorporated the random effect of successive segments from a single dolphin focal group. Models were run using the multiple generalized cross-validation (mgcv) package in program R [73] appropriate for detecting trends in complex data that are multivariate and nonlinear [58]. Generalized additive models incorporate smoothing terms, fitting data locally rather than globally [67], with a penalty for excessive flexibility [73, 74]. Flexibility was determined by the number of knots for each smooth term. The default value of 10 knots, set by package mgcv, was used unless there were fewer than 10 categories per term, in which case the knot value was lowered. Models were tested with all combinations of the fixed factors and Akaike Information Criterion correction (AICc) values were calculated and compared. The AICc is derived from AIC and is appropriate for smaller datasets where n<40 data records per parameter [75, 76]. These models evaluate candidate explanatory variables simultaneously, reducing problems associated with many step-wise techniques. Model selection was based on adj-R^2^ (high), GCV (low), and deviance explained (high).

Microsoft Excel (v 2013) was used to conduct computational analysis of swimming speed, reorientation rate, and linearity and to calculate log-likelihood and binomial *z* score statistics; R statistical software was used to perform exploratory work, autocorrelation tests, and GAM analyses; and ArcGIS software (v 10.2.2) from Environmental Systems Research Institute (ESRI) was used to produce the map.

## Results

### Behavioral activity states

**Fig 2:**
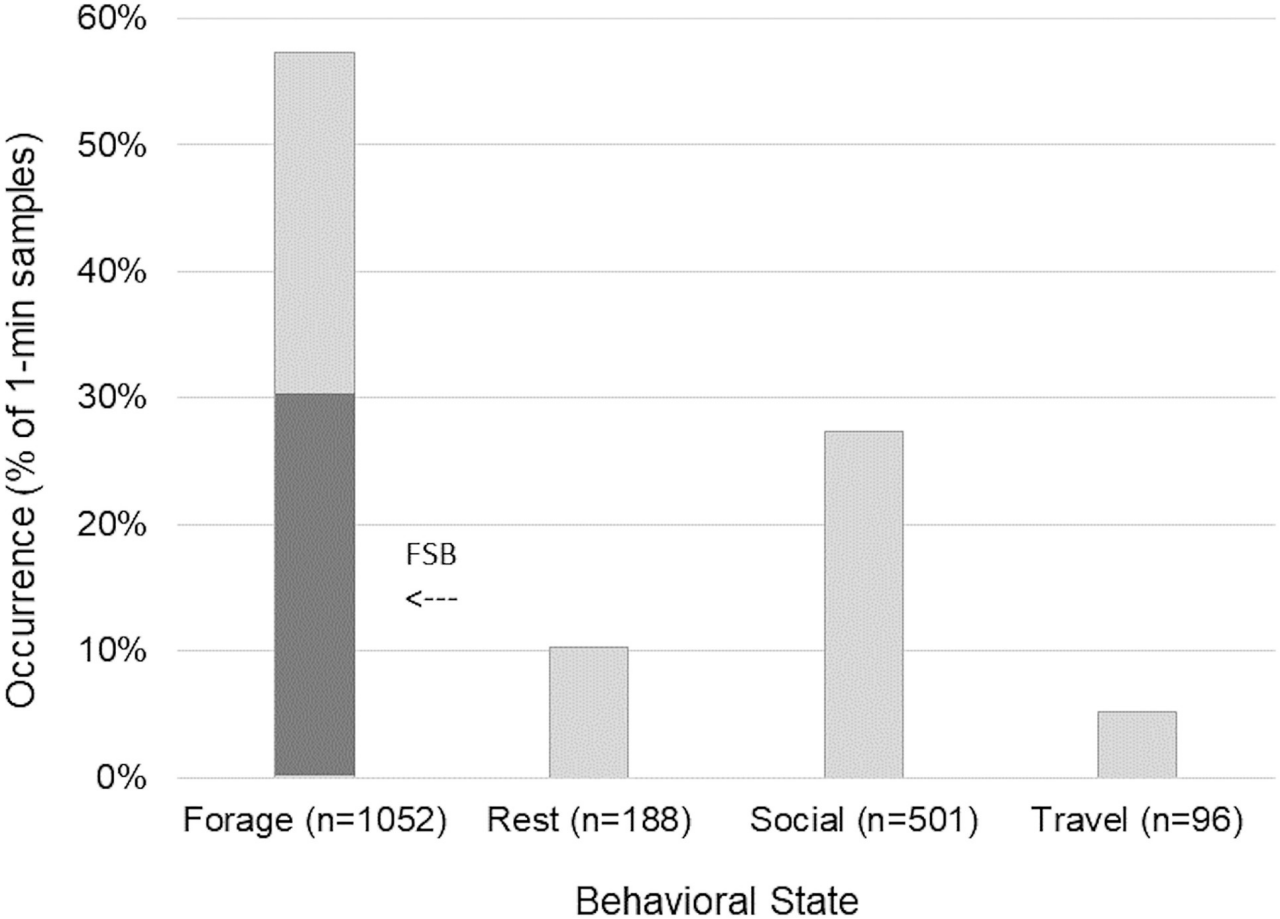
Behavioral activity states of bottlenose dolphin groups in the Galveston Ship Channel. FSB indicates foraging in association with shrimp trawlers.

Observed dolphin behavioral states varied significantly from values expected by chance (Chi-square test, χ^2^ = 1216.38, n = 1,837, df = 3, P<0.001) with foraging (57%, n = 1052) and socializing (27%, n = 501) observed more than expected, and travelling (5%, n = 96) and resting (10%, n = 188) observed less than expected (Fig 2). Foraging in association with trawlers and without trawlers accounted for 30% and 27%, respectively, of observed behavior. Foraging categories did not vary significantly (Chi-square test, χ^2^ = 2.77, n = 1,052, df = 1, P<0.05).

**Fig 3:**
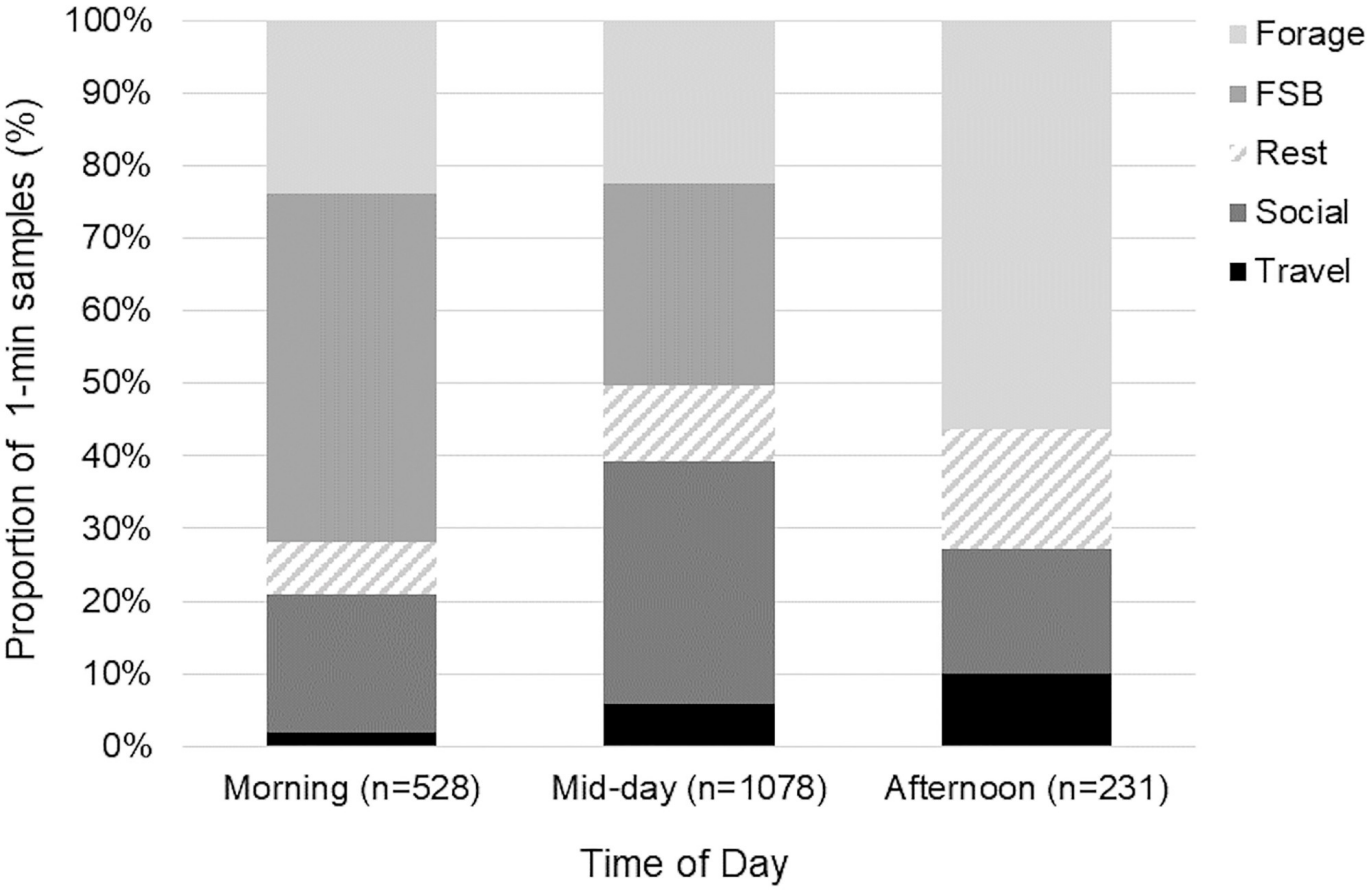
Bottlenose dolphin behavioral activity states based on time of day. Percentile from sunrise (0) to sunset (1) in which morning = 0–0.33, mid-day = 0.34–0.66, and afternoon = 0.67–1. FSB indicates foraging in association with shrimp trawlers.

Dolphin behavior varied significantly by time of day (log-likelihood ratio test, G^2^ = 328.13, n = 1,837, df = 8, P<0.001; Fig 3). Post hoc tests showed that, compared to other times of day, dolphins were more likely to be foraging in association with trawlers during the morning (*z* = 8.84), and more likely to be socializing during mid-day (*z* = 5.99). During the afternoon, dolphins were more likely to be foraging without trawlers (*z* = 9.08) than during mid-day, and more likely to be resting (*z* = 3.16) and traveling (*z* = 3.36) than in the morning.

**Fig 4:**
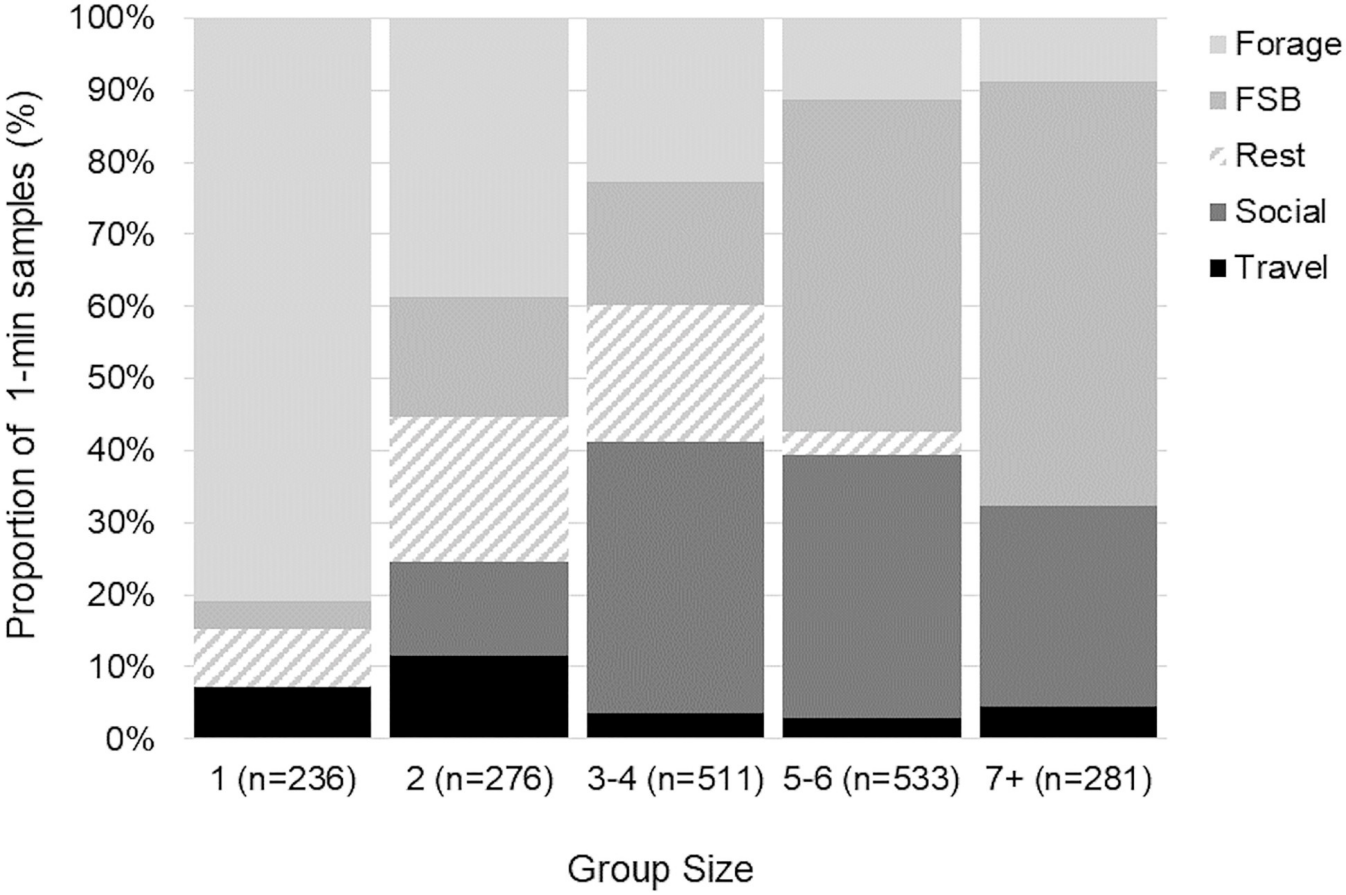
Bottlenose dolphin behavioral activity states based on group size. FSB indicates foraging in association with shrimp trawlers.

Dolphin behavior varied significantly with group size (G^2^ = 888.49, n = 1,837, df = 16, P<0.001; Fig 4). The mean group size was 3.85 dolphins (SD = ±2.21) with a range of 1–12 dolphins, similar to prior findings from the early 1990’s with a range of 1–15 dolphins [15]. Post hoc tests showed, in general, if a sample occurred in larger groups, dolphins were more likely to be foraging in association with trawlers (*z* = 19.07, $\bar{\text{x}}=5.68\pm \text{SD}\;2.45$), or socializing (*z* = 14.57, $\bar{\text{x}}=4.89\pm \text{SD}\;1.80$), than in smaller groups. In smaller groups, foraging without trawlers was more likely (*z* = 16.98, $\bar{\text{x}}=2.66\pm \text{SD}\;2.02$). In groups of 2–4 individuals, resting was more likely (*z* = 7.28, $\bar{\text{x}}=2.89\pm \text{SD}\;1.15$), and if a sample occurred in groups of 2, traveling was more likely (*z* = 5.02, $\bar{\text{x}}=3.50\pm \text{SD}\;2.40$).

**Fig 5:**
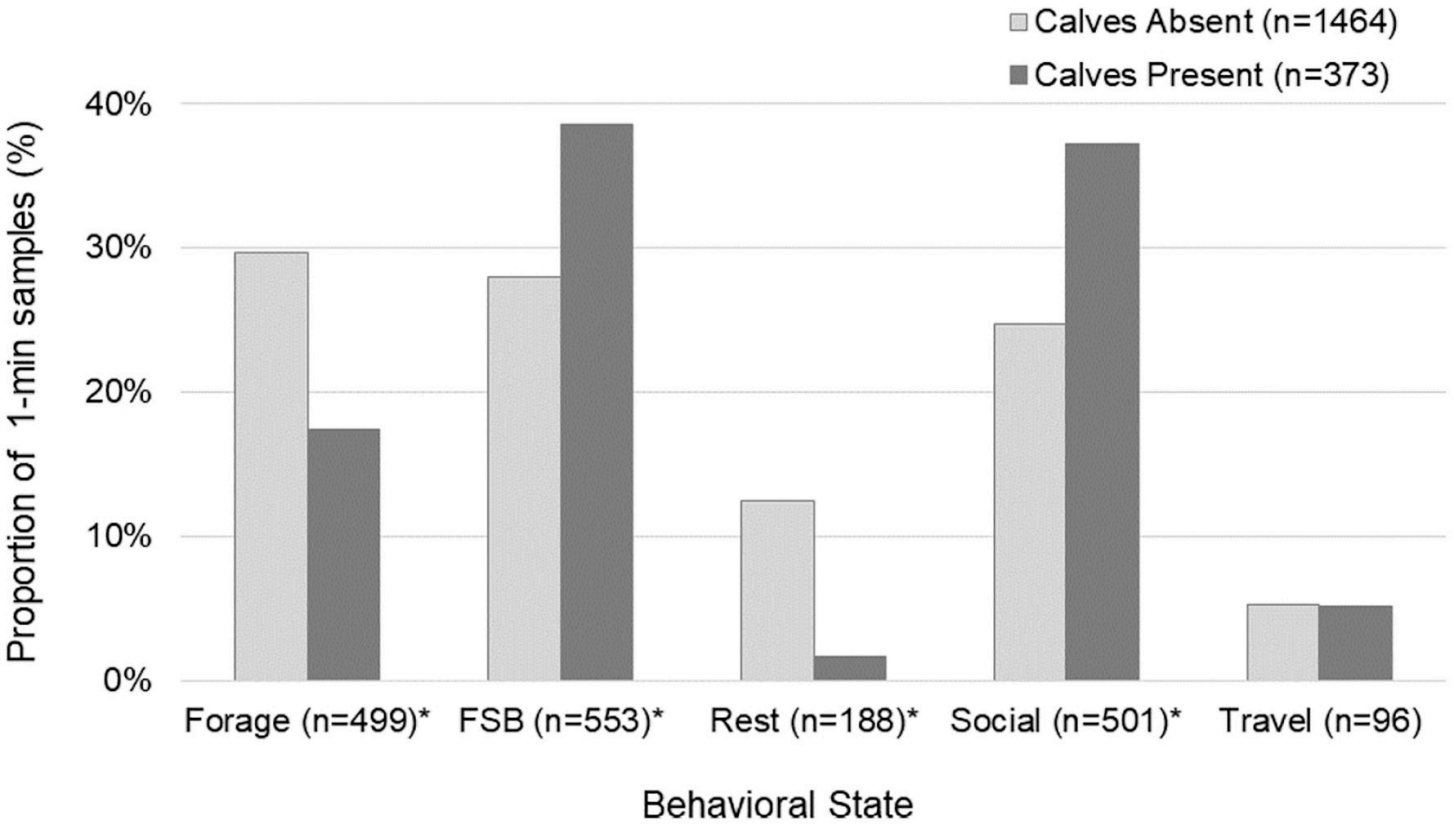
Bottlenose dolphin behavioral activity states based on calf presence. FSB indicates foraging in association with shrimp trawlers. Asterisk indicates statistically significant difference.

At least one calf was present in 20% (n = 373) of the groups tracked. Dolphin behavior varied significantly with calf presence (log-likelihood ratio test, G^2^ = 93.162, n = 1,837, df = 4, P<0.001; Fig 5). Post hoc tests showed that if calves were present in a sample, dolphins were more likely to be socializing (*z* = 4.14) or foraging in association with trawlers (*z* = 3.35) and less likely to be resting (*z* = -5.83) or foraging without trawlers (*z* = -4.04).

**Fig 6:**
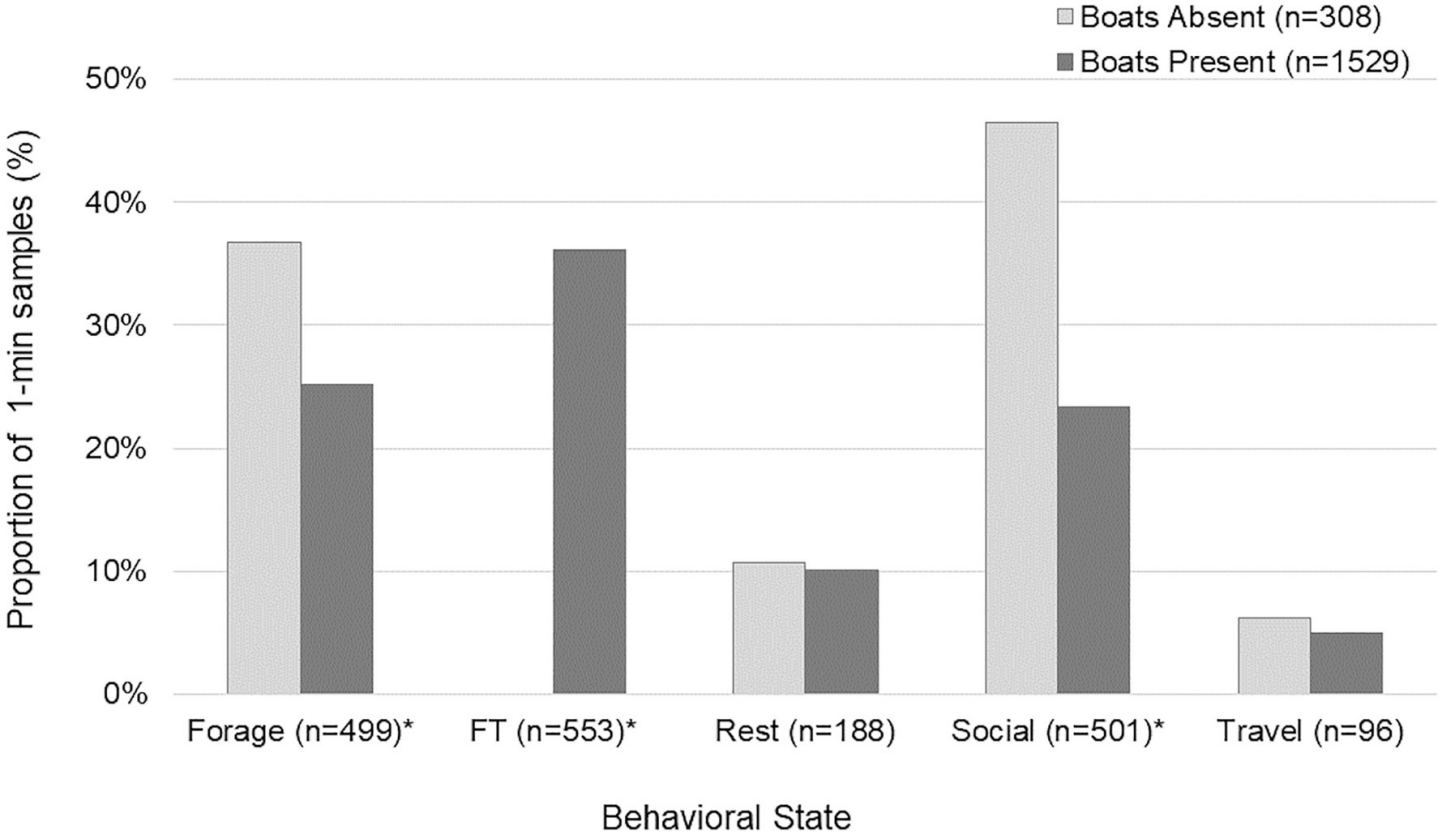
Bottlenose dolphin behavioral activity states based on vessel presence. Asterisks indicates statistically significant differences.

Dolphin behavior varied significantly with vessel presence (G^2^ = 257.97, n = 1,837, df = 4, P<0.001; Fig 6). Post hoc tests showed that if vessels were present, dolphins were more likely to be foraging in association with trawlers (*z* = 10.55), and less likely to be socializing (*z* = -7.06) or foraging without trawlers (*z* = -3.52). Vessels were closer than 45m from dolphins, the NMFS recommended minimum viewing distance, during 42% of unfiltered tracks, including 21 accounts of boats transiting directly through dolphin groups.

### Fine-scale movement patterns

#### Swimming speed

The GAM described significant variation in swimming speed, at the 0.05 alpha level, explaining 45.3% of the deviance (adj-R^2^ = 0.390, GCV = 0.054, n = 167). The best fitting model included all five candidate explanatory variables with a smooth term for both time of day and dolphin group size, and linear terms for calf presence, predominant group behavior, and vessel category:

[Log10(Speed)~s(TimeOfDay)+s(GrpSize)+Calf+BehavState+VesselCat]

**Fig 7:**
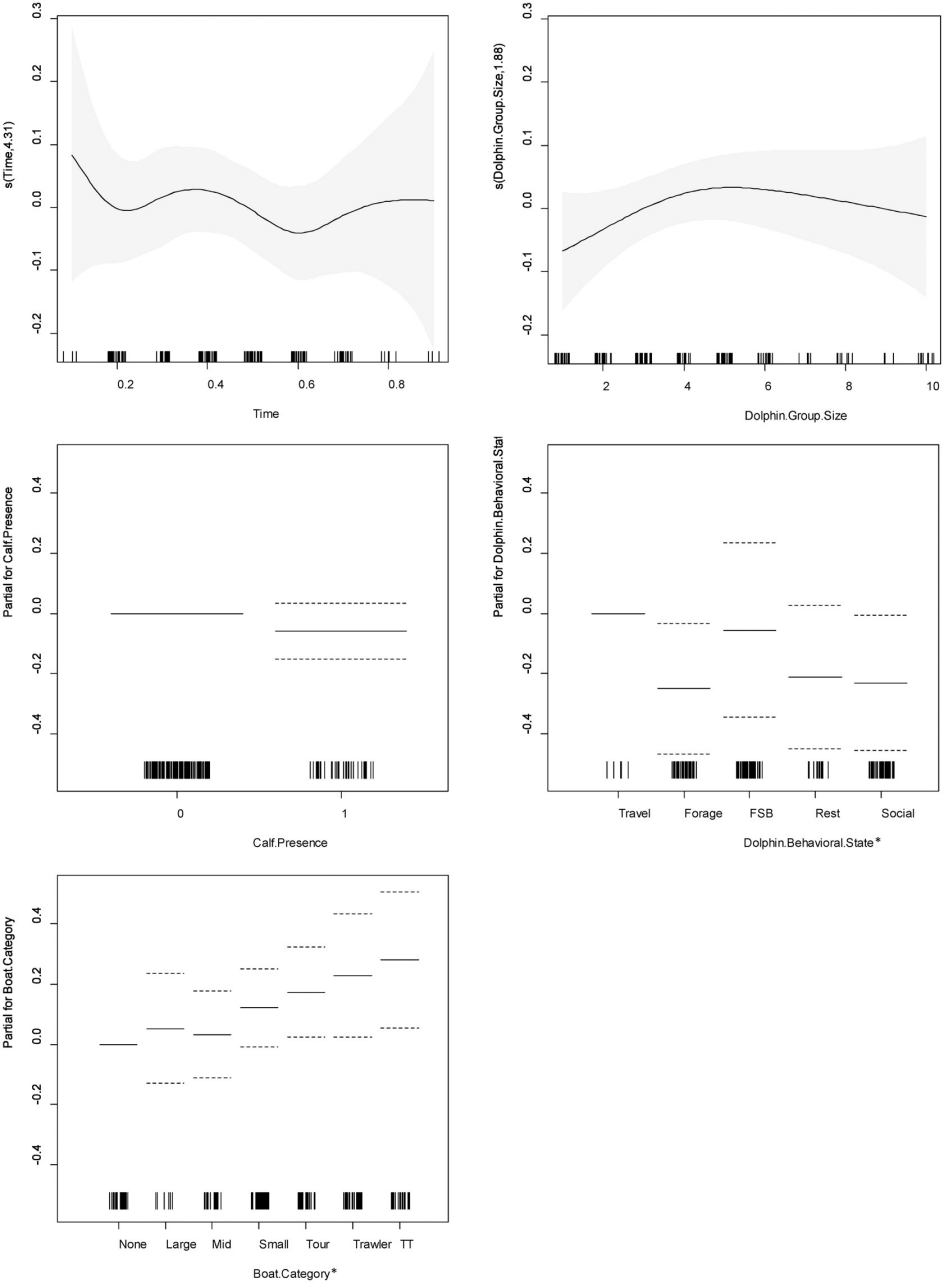
Charts for the partial contribution of individual explanatory variables in the fitted GAM for swimming speed. Includes A) time of day, B) dolphin group size, C) calf presence, D) dolphin behavioral state, and E) vessel category. The rugplot along the x-axis indicates the number of observations for each factor. The gray shading for smooth terms, and the dotted lines for linear terms, indicate the 95% confidence intervals. On the y-axis, values >0 indicate a positive correlation with swimming speed, values <0 indicate a negative correlation, and a value of 0 indicates no effect. An asterisk (*) indicates a variable with a statistically significant effect at alpha level 0.05.

Swimming speed was significantly higher in the presence of tour boats, trawlers, and highest when both tour boats and trawlers were present during the same sampling interval (i.e., tour boats follow dolphins that are following trawlers). Travelling behavior was associated with significantly higher swimming speed along the horizontal plane than foraging and socializing. At the 0.1 alpha level, swimming speed was significantly higher in the presence of small recreational boats and travelling was associated with significantly higher speed than resting. Time of day, dolphin group size and calf presence had no significant effect on swimming speed, though there was variation based on these factors (S1 Table; Fig 7).

#### Reorientation rate

The GAM described significant variation in reorientation rate, at the 0.05 alpha level, explaining 42.4% of the deviance (adj-R^2^ = 0.361, GCV = 3.440, n = 167). The best fitting model included all five candidate explanatory variables with a smooth term for both time of day and dolphin group size, and linear terms for calf presence, predominant group behavior, and vessel category:

[Sqrt(ReorientationRate)~s(TimeOfDay)+s(GrpSize)+Calf+BehavState+VesselCat]

**Fig 8:**
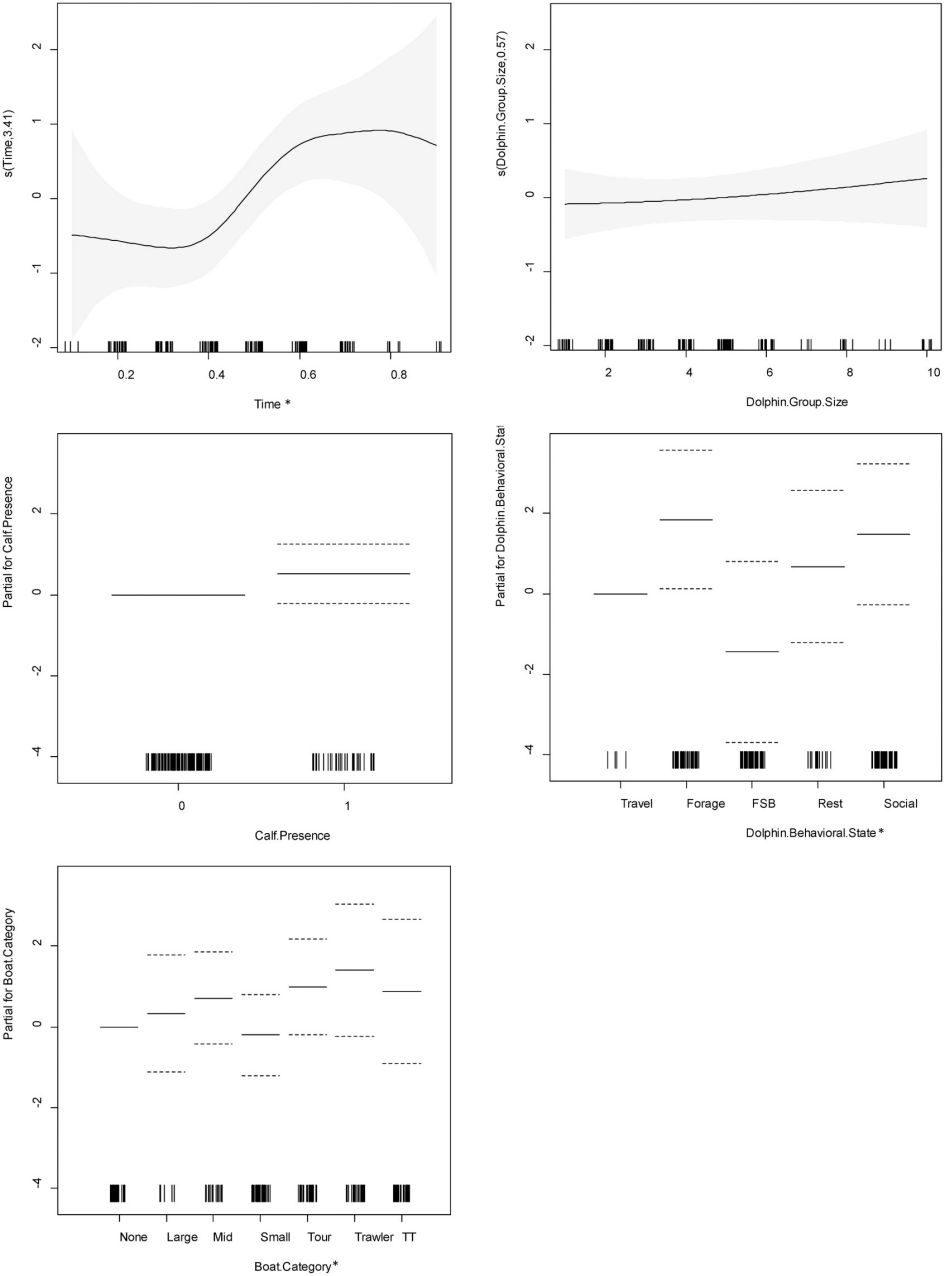
Charts for the partial contribution of individual explanatory variables in the fitted GAM for reorientation rate. Includes A) time of day, B) dolphin group size, C) calf presence, D) dolphin behavioral state, and E) vessel category. The rugplot along the x-axis indicates the number of observations for each factor. The gray shading for smooth terms, and the dotted lines for linear terms, indicate the 95% confidence intervals. On the y-axis, values >0 indicate a positive correlation with reorientation rate, values <0 indicate a negative correlation, and a value of 0 indicates no effect. An asterisk (*) indicates a variable with a statistically significant effect at alpha level 0.05. A closed circle (•) indicates a variable with a statistically significant effect at alpha level 0.1.

The non-linear relationship between reorientation rate and time of day was significant, with the lowest reorientation rates occurring from late morning to mid-day and the highest reorientation rates occurring from the afternoon to early evening. Travelling behavior was associated with significantly lower reorientation rates than foraging behavior. At the 0.1 alpha level, travelling was associated with lower reorientation rates than socializing, and dolphins reoriented significantly more in the presence of tour boats and trawlers. Dolphin group size and calf presence had no significant effect on reorientation rate (S2 Table; Fig 8).

#### Linearity

The GAM described significant variation in linearity, at the 0.05 alpha level, explaining 31.5% of the deviance (adj-R^2^ = 0.230, GCV = 4.058, n = 167). The best fitting model included all five candidate explanatory variables with a smooth term for both time of day and dolphin group size, and linear terms for calf presence, predominant group behavior, and vessel category:

[EmpLogit(Linearity)~s(TimeOfDay)+s(GrpSize)+Calf+BehavState+VesselCat]

**Fig 9:**
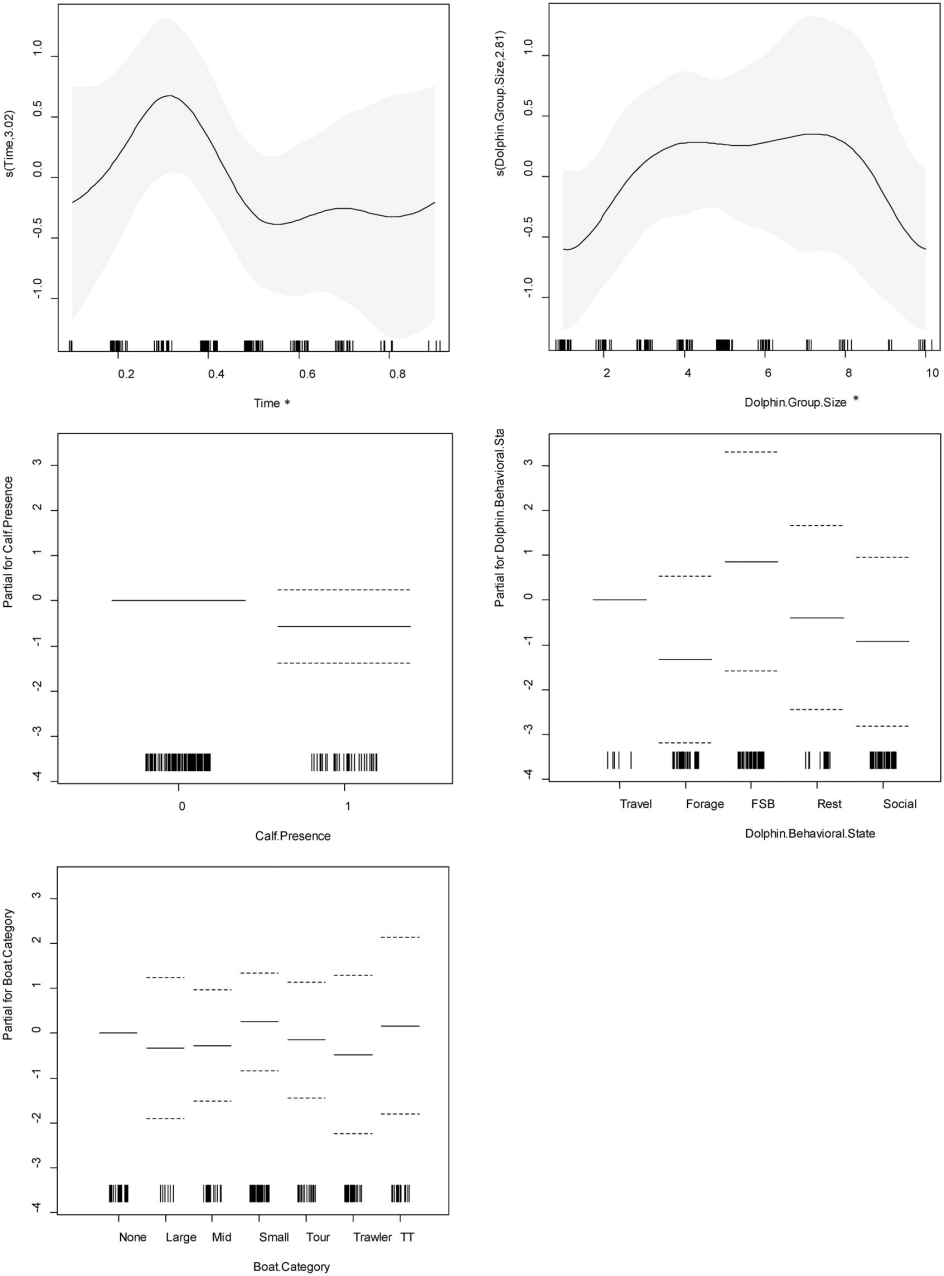
Charts for the partial contribution of individual explanatory variables in the fitted GAM for linearity. Includes A) time of day, B) dolphin group size, C) calf presence, D) dolphin behavioral state, and E) vessel category. The rugplot along the x-axis indicates the number of observations for each factor. The gray shading for smooth terms, and the dotted lines for linear terms, indicate the 95% confidence intervals. On the y-axis, values >0 indicate a positive correlation with linearity, values <0 indicate a negative correlation, and a value of 0 indicates no effect. An asterisk (*) indicates a variable with a statistically significant effect at alpha level 0.05. A closed circle (•) indicates a variable with a statistically significant effect at alpha level 0.1.

The non-linear relationship between linearity and time of day indicated more linear movement from late morning to mid-day and less linear movement from afternoon to early evening, which is congruent with reorientation rate patterns. The relationship between linearity and dolphin group size indicated more linear movement in groups of three to eight dolphins, and less linear movement in smaller and larger groups. Vessel category, calf presence, and dolphin behavior had no significant effect on linearity (S3 Table; Fig 9).

## Discussion

This study highlights variability in dolphin behavior and movement patterns relative to endogenous and exogenous factors and vessel activity in a narrow seaport. Bottlenose dolphins occur regularly in the GSC during summer months. They were observed every sampled day, during all hours of daylight and during periods of low and high vessel traffic. Dolphins use the GSC primarily for foraging and socializing, and it does not appear to be a travel corridor to access other favorable sites.

Diurnal behavioral activity states were generally congruent with an early 1990’s study in the Galveston Bay system and adjacent coastal Gulf of Mexico waters [77] in which traveling behavior peaked in the afternoon and socializing increased as foraging behavior decreased. Dolphins presumably travelled in the afternoon to return to Galveston Bay from coastal gulf waters. Post-feeding socializing has also been observed in other cetacean species that include Guiana dolphins, *Sotalia guianensis*, [78] and dusky dolphins (*Lagenorhynchus obscurus*) off Argentina [79]. In this study, socializing was observed significantly more in larger groups. Socializing encompasses a variety of events, including active socio-sexual displays at the water’s surface and less obvious interactions, such as pectoral fin rubbing between two individuals. It is possible that there was a sighting bias towards more obvious displays that included larger numbers of active individuals. Resting and travelling were observed significantly more in smaller groups. Group formation may reduce susceptibility to predation and enhance detection and capture of prey that are patchily distributed [80]. However, when resources are predictable and found in complex inshore environments where predator density is low, there may be few benefits to large group formation [80]. This might explain why dolphins in the GSC form smaller groups when engaged in resting, travelling, and foraging independent of trawlers.

Dolphin behavioral states and movement patterns varied significantly when vessels were in close proximity. These responses to vessels may result in increased energy expenditure (e.g., increased swimming speed) and decreased energy consumption (e.g., disrupted foraging activity) that may result in overall reduced energy acquisition [81]. This is especially important when considering lactating females and dependent calves, given that lactation is one of the most energetically expensive periods for female mammals [82]. Reduced foraging and socializing behavior due to human activity has also been described in other dolphin studies [33, 83]. Furthermore, dolphins in the Galveston Bay and GSC area occur in relatively small numbers, with some individual site fidelity [15], increasing the likelihood of repeated exposure of individual dolphins to vessels.

Distinct vessel type affected dolphins in different ways. The sample size for the large vessel category was low and it is likely that these slow, predictable linear-moving vessels were relatively easy for dolphins to detect and avoid. Vessels categorized as mid-sized were also relatively slow and linear in movement, compared to vessels categorized as small that moved quickly and unpredictably, at times. The narrow, active GSC may restrict opportunities for lateral movement, and dolphins may not have adequate time to avoid fast boats that move in a non-linear path, as indicated by encounters of small boats operating directly through focal groups. Field observations suggest that dolphins may alter behavior along the vertical water column, as inter-surfacing intervals appear to increase in the presence of small boats. Several other studies have shown an increase in breathing synchrony and longer inter-breath intervals of dolphins in response to vessels [38, 84, 85]. Mother-calf pairs may be especially vulnerable to this type of boat activity because calves are not fully developed and are physiologically limited in their ability to swim and dive [86, 87], which may hinder maneuverability of both mother and calf. This behavior was difficult to quantify while tracking groups of dolphins from land using a theodolite that tracks movements along the horizontal water surface. Additional data collection on individual surfacing intervals in the presence of small, fast-moving boats is suggested to address this stressor. There is currently a recommended “no wake” speed (no more than approximately 8km/hr) in the GSC, but few adhere to it (e.g., small recreation boats were recorded travelling up to 79km/hr). Establishing a formal speed limit in the GSC may help reduce small recreational boating disturbance to dolphins.

Fifty-three percent of the recorded foraging activity was in association with trawlers. Odontocetes are often oriented around fishing vessels where acquisition of concentrated prey sources, and prey that are disoriented or injured, may be facilitated [3]. Dolphin groups that foraged in association with trawlers were larger than those foraging without trawlers. This is incongruent with results of prior research in the GSC in which the smallest groups foraged behind trawlers ($\bar{\text{x}}=2.70\pm \text{SD}\;1.78$) [15]. During the present study, groups with calves foraged in association with trawlers significantly more than foraging without trawlers, which may reflect the high energetic needs of breeding females [3]. The same pattern has been observed in other populations. For example, off Hong Kong, humpback dolphin groups with calves were more likely to feed near fishing vessels than those without calves [88]. Social learning over the years may contribute to the shift in group size associated with trawlers in the GSC, especially considering a higher percentage of groups with calves engage in this type of foraging than those foraging without trawlers. Dolphins significantly altered their patterns of movement and behavior to more closely match the movement patterns of trawlers, highlighting alternative tactics for hunting prey in response to human activity. In Australia, humpback dolphins (*Sousa sahulensis*) feed in association with fishing trawlers as a major source of food [89], and Indo-Pacific humpback dolphins (*S*. *chinensis*) off Hong Kong fed near commercial trawlers when trawling operations there were still active [88, 90]. There appeared to be inter-individual selection for this type of feeding in both populations. It is unclear if a similar individual or community preference exists in the GSC population. However, one study in the early 1990’s showed that 74% of photo-identified individuals were sighted at least once in association with trawlers [16]. Delineating present day community structure and foraging tactics in the overall population will offer additional information on this specialized form of foraging.

Commercial marine mammal tourism has often been considered a benign and sustainable activity, especially as an alternative to directed hunting. However, the proliferation of the industry on a global scale in recent decades raises questions about potential effects on target populations [91, 92]. This study shows that dolphin-watching tourism in the GSC can lead to behavioral harassment. These short-term responses to vessels can cause shifts in behavior and habitat-use (e.g., reduced foraging and socializing and increased travelling), reduced energy consumption (e.g., disrupted foraging time), and interference with conspecific communication. These short-term changes do not necessarily affect long-term individual health and survival or population viability. However, dolphins that express repeated responses to a stimulus over time may experience increased energetic expenditure and chronic stress with broader biological, physiological and/or ecological consequences [32, 93]. For example, boat-based tourism was negatively correlated with female bottlenose dolphin reproductive success off Australia in an area of long-term tourism disturbance [53]. In the GSC, there are currently three tour operations that advertise dolphin viewing (other boat-based businesses view dolphins opportunistically), one of which began after this research concluded, with no regulatory or management framework in place. Without proper management, short-term changes may lead to long-term consequences not only to individuals, but the entire population [32].

## Conclusions and recommendations

Findings from this research show that some vessel activity in the GSC affects dolphin behavior and movement patterns, including small boats, commercial trawlers, and dolphin-watching tourism boats. Dolphins showed behavioral flexibility in exploiting food resources in which prey acquisition is facilitated by commercial trawlers. However, dolphins may be at greater risk of being struck by a vessel or incurring propeller lacerations when in close proximity. Risk may be elevated when tour vessels follow dolphins that feed in association with trawlers, at which point dolphins become entrapped between at least two vessels during an energy-enhancing activity. It is unclear if this risky attractant has positive, neutral, or negative consequences to the overall population. No behavioral responses were detected in the presence of large and mid-sized vessels and they do not appear to be a behavioral stressor to dolphins in the GSC. However, data were lacking for physiological changes and potential internal responses could not be detected.

In many regions where dolphins and humans overlap, few behavior-sensitive regulatory or management frameworks exist, and especially lacking are plans for long-term management [94]. There are currently no permit requirements for dolphin-watching tourism in the U.S. Southeast Region [95]. The National Oceanic and Atmospheric Administration (NOAA) has a voluntary code of guidelines for viewing dolphins in the Southeast Region, which aims to reduce the potential for harassment. Unfortunately, voluntary codes of conduct for dolphin watching, though well-intended, are often inadequate without hands-on practical operator training and guidance. For example, tour boats were observed operating contrary to NOAA recommendations, including breaching the minimum viewing distance of 45m (at times following dolphins at less than 2m), encircling/entrapping dolphins between their boat and another boat, making sudden changes in speed and direction in the vicinity of dolphins, approaching dolphins head-on, and approaching dolphins when another vessel was in proximity. Current legislation and recommended viewing guidelines do not protect dolphins in the GSC from behavioral harassment, as shown in this study.

In the U.S., Marine Mammal Protection Act permits are required to conduct boat- and air-based research and education, and require descriptions of operator experience around marine mammals, adherence to guidelines, and annual reporting, including estimated number of dolphins approached. This is an excellent way to manage the number of researchers/educators in a given area, and to limit the number of animals that are approached with potential for harassment. A similar mandatory permitting requirement for commercial tour operations may minimize disturbance to dolphins. The New Zealand Department of Conservation serves as an example of how a formal permitting process aids in managing the rapidly growing commercial marine mammal tourism industry and aims to minimize effects on marine mammal behavior. Research-based recommendations are often integrated to assist in informing regulatory and management decisions. Responsible wildlife viewing can stimulate local economies and promote public interest in dolphin conservation in positive ways, but operations should be conducted with appropriate knowledge of dolphin behavior. Regulating and monitoring dolphin-watching tourism in the GSC, with formal permit requirements, would help to ensure that it is sustainable and operating within the principles of the MMPA. In narrow seaports like the GSC, dolphins regularly occur very close to shore and land-based viewing is an excellent non-invasive alternative to boat-based viewing.

Dolphins do not appear to abandon the GSC, even during periods of consistent and intensive vessel presence in which boats actively follow dolphins. This may be due, in part, to lack of ecologically similar habitat, with similar prey characteristics, proximate to the GSC. Dolphins may not avoid high risk areas that support key resources such as prey, particularly if adjacent habitats are not equivalent [96]. Dolphins in the GSC may benefit from an adaptive management scheme that evolves based on ongoing review of management goals and methods are adjusted as new information is obtained [97, 98]. This iterative process includes several steps: planning, implementing, monitoring, reviewing, learning, revising, and repeating [97], and may be especially important in dynamic environments with shifting environmental and anthropogenic input. Adaptive management benefits from rapid implementation of revised protocols but may be more challenging in developed countries with structured top-down legislative systems [97]. Findings from this study can be broadly informative to areas where delphinids and vessels overlap, and more specifically, where delphinids occur in narrow, deep channels that support concentrated vessel traffic.

## Supporting information

S1 Fig Augmented pairs plot with potential explanatory factors: Time (timeper), group size (grpsize), calf presence (calf), behavior (behave), type of boats (cat), and number of boats (numboat) present. The only factors that showed potential collinearity were type of boats and number of boats at 0.65. The number of boats factor was eliminated from the dataset to reduce potential masking effects associated with collinearity.
(TIF)

S2 Fig Autocorrelation structure for swimming speed (left), reorientation rate (middle), and linearity (right) after filtering data. No autocorrelation was detected in the residuals.
(TIF)

S1 Table Summary of output for best fitting model for bottlenose dolphin swimming speed. (DOCX)

S2 Table Summary of output for best fitting model for bottlenose dolphin reorientation rate. (DOCX)

S3 Table Summary of output for best fitting model for bottlenose dolphin linearity. (DOCX)
