# Supplementary figures and images for: Common bottlenose dolphin (Tursiops truncatus) behavior in an active narrow seaport
Source: PLoS One. 2019 Feb 19;14(2):e0211971. doi: 10.1371/journal.pone.0211971 (PMC6380569; doi:10.1371/journal.pone.0211971)

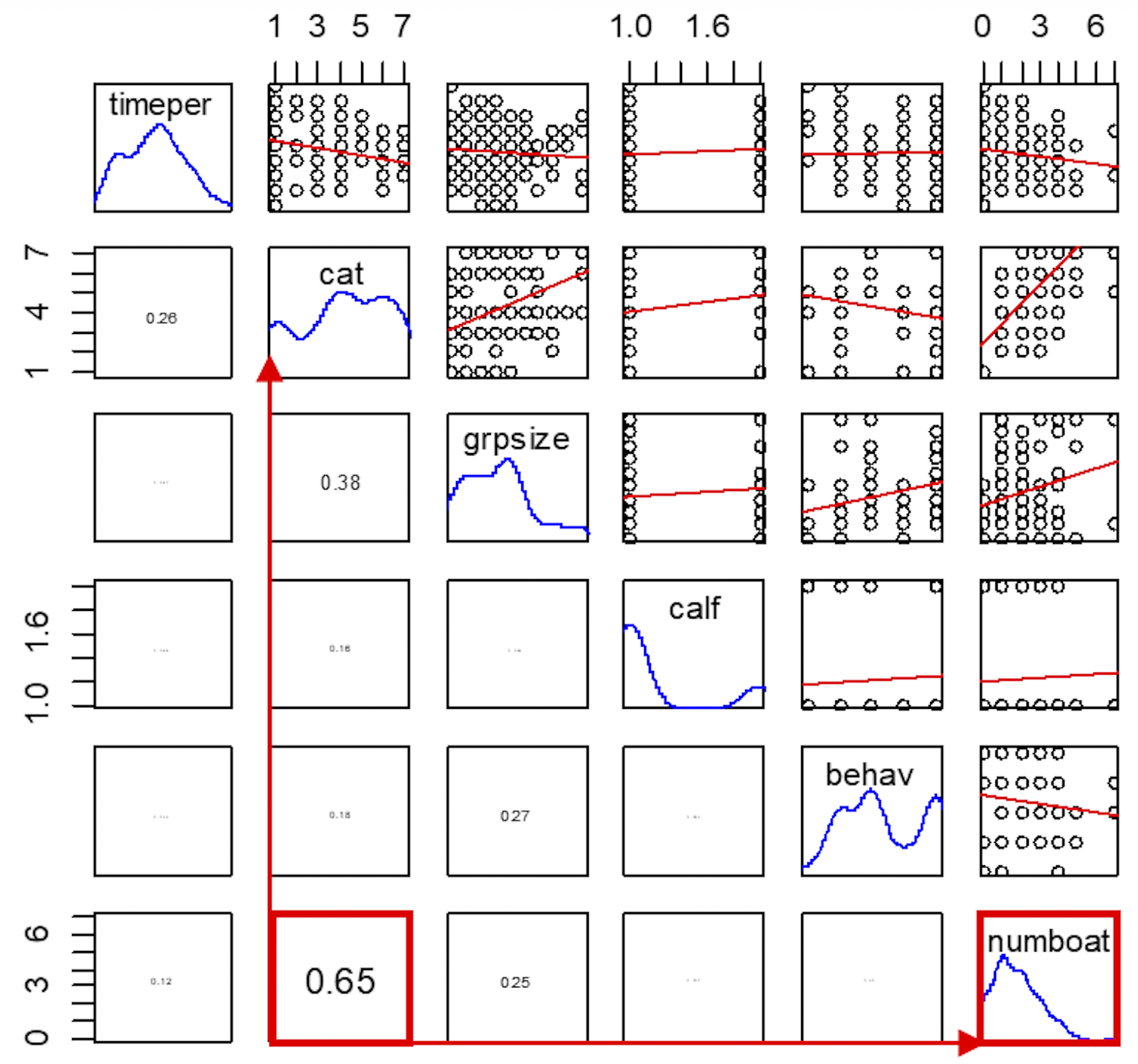

Supplement: S1 Fig — The only factors that showed potential collinearity were type of boats and number of boats at 0.65. The number of boats factor was eliminated from the dataset to reduce potential masking effects associated with collinearity. (TIF) [file pone.0211971.s001.tif]

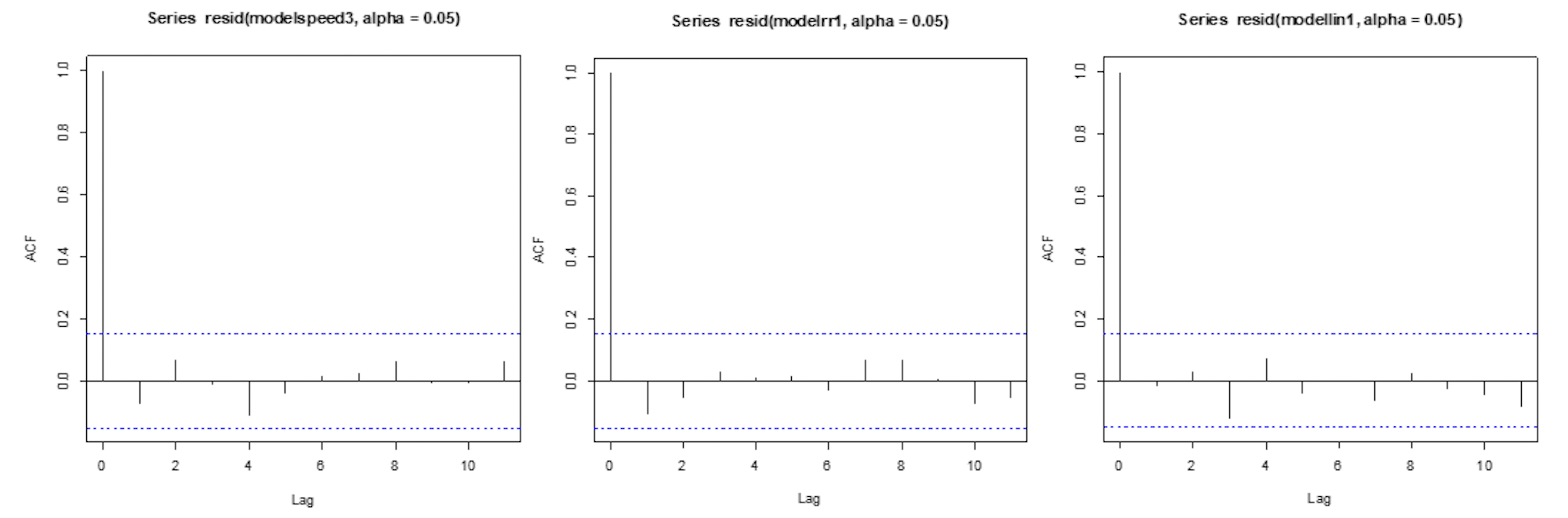

Supplement: S2 Fig — No autocorrelation was detected in the residuals. (TIF) [file pone.0211971.s002.TIF]
