# Supplementary material for: Common bottlenose dolphin (Tursiops truncatus) behavior in an active narrow seaport
Source: PLoS One. 2019 Feb 19;14(2):e0211971. doi: 10.1371/journal.pone.0211971 (PMC6380569; doi:10.1371/journal.pone.0211971)
Supplement: S1 Table — (DOCX) [file pone.0211971.s003.docx]

**S1 Table.**

| Term | Estimate | Std. Error | T | *P*-value |
| --- | --- | --- | --- | --- |
| (Intercept) | 0.327 | 0.117 | 2.789 | 0.006 |
| Calf – Present | -0.058 | 0.047 | -1.253 | 0.212 |
| BehavState – Foraging | -0.250 | 0.108 | -2.318 | 0.022* |
| BehavState – FSB | -0.055 | 0.145 | -0.383 | 0.703 |
| BehavState – Resting | -0.211 | 0.119 | -1.781 | 0.077 • |
| BehavState - Socializing | -0.231 | 0.112 | -2.062 | 0.041* |
| VesselCat – Large | 0.052 | 0.091 | 0.570 | 0.570 |
| VesselCat – Mid | 0.033 | 0.072 | 0.454 | 0.651 |
| VesselCat – Small | 0.121 | 0.064 | 1.882 | 0.062 • |
| VesselCat – Tour | 0.173 | 0.075 | 2.325 | 0.021* |
| VesselCat – Trawler | 0.228 | 0.102 | 2.235 | 0.027* |
| VesselCat - Tour&Trawler | 0.280 | 0.113 | 2.477 | 0.014* |
|  | Edf |  | F | *P*-value |
| s(TimeOfDay) | 4.314 |  | 0.534 | 0.776 |
| s(GrpSize) | 1.879 |  | 1.131 | 0.281 |

Includes linear (top) and smooth (bottom) terms. Linear categorical terms are estimated relative to the reference value for that term: Absent (calf), Travelling (behavioral state), and None (vessel category).

*Indicates a variable with a statistically significant effect at alpha level 0.05.

•Indicates a variable with a statistically significant effect at alpha level 0.1.
