# Supplementary material for: Common bottlenose dolphin (Tursiops truncatus) behavior in an active narrow seaport
Source: PLoS One. 2019 Feb 19;14(2):e0211971. doi: 10.1371/journal.pone.0211971 (PMC6380569; doi:10.1371/journal.pone.0211971)
Supplement: S2 Table — (DOCX) [file pone.0211971.s004.docx]

**S2 Table.**

| Term | Estimate | Std. Error | T | *P*-value |
| --- | --- | --- | --- | --- |
| (Intercept) | 4.825 | 0.916 | 5.267 |  |
| Calf – Present | 0.524 | 0.365 | 1.434 | 0.154 |
| BehavState - Foraging | 1.840 | 0.862 | 2.134 | 0.035 * |
| BehavState - FSB | -1.443 | 1.123 | -1.285 | 0.201 |
| BehavState - Resting | 0.672 | 0.944 | 0.711 | 0.478 |
| BehavState - Socializing | 1.474 | 0.873 | 1.687 | 0.094 • |
| VesselCat – Large | 0.326 | 0.725 | 0.449 | 0.654 |
| VesselCat - Mid | 0.713 | 0.569 | 1.254 | 0.212 |
| VesselCat - Small | -0.197 | 0.503 | -0.392 | 0.695 |
| VesselCat - Tour | 0.989 | 0.590 | 1.677 | 0.096 • |
| VesselCat - Trawler | 1.397 | 0.814 | 1.716 | 0.088 • |
| VesselCat - Tour&Trawler | 0.877 | 0.892 | 0.983 | 0.327 |
|  | Edf |  | F | *P*-value |
| s(TimeOfDay) | 3.407 |  | 3.669 | 0.006 * |
| s(GrpSize) | 0.572 |  | 0.518 | 0.490 |

Includes linear (top) and smooth (bottom) terms. Linear categorical terms are estimated relative to the reference value for that term: Absent (calf), Travelling (behavioral state), and None (vessel category).

*Indicates a variable with a statistically significant effect at alpha level 0.05.

•Indicates a variable with a statistically significant effect at alpha level 0.1.
