# Supplementary material for: Common bottlenose dolphin (Tursiops truncatus) behavior in an active narrow seaport
Source: PLoS One. 2019 Feb 19;14(2):e0211971. doi: 10.1371/journal.pone.0211971 (PMC6380569; doi:10.1371/journal.pone.0211971)
Supplement: S3 Table — (DOCX) [file pone.0211971.s005.docx]

**S3 Table.**

| Term | Estimate | Std. Error | T | *P*-value |
| --- | --- | --- | --- | --- |
| (Intercept) | 1.133 | 0.965 | 1.174 | 0.242 |
| Calf – Present | -0.523 | 0.405 | -1.292 | 0.198 |
| BehavState - Foraging | -1.289 | 0.923 | -1.397 | 0.165 |
| BehavState - FSB | 0.780 | 1.212 | 0.643 | 0.521 |
| BehavState - Resting | -0.437 | 1.022 | -0.428 | 0.670 |
| BehavState - Socializing | -0.941 | 0.942 | -0.999 | 0.319 |
| VesselCat – Large | -0.367 | 0.780 | -0.471 | 0.638 |
| VesselCat - Mid | -0.262 | 0.618 | -0.424 | 0.672 |
| VesselCat - Small | 0.280 | 0.541 | 0.517 | 0.606 |
| VesselCat - Tour | -0.172 | 0.647 | -0.266 | 0.791 |
| VesselCat - Trawler | -0.429 | 0.874 | -0.491 | 0.624 |
| VesselCat - Tour&Trawler | 0.144 | 0.981 | 0.147 | 0.883 |
|  | Edf |  | F | *P*-value |
| s(TimeOfDay) | 3.067 |  | 1.070 | 0.043 * |
| s(GrpSize) | 4.253 |  | 1.399 | 0.080 • |

Includes linear (top) and smooth (bottom) terms. Linear categorical terms are estimated relative to the reference value for that term: Absent (calf), Travelling (behavioral state), and None (vessel category).

*Indicates a variable with a statistically significant effect at alpha level 0.05.

•Indicates a variable with a statistically significant effect at alpha level 0.1.
